# Metabolic Reprogramming Into a Glycolysis Phenotype Induced by Extracellular Vesicles Derived From Prostate Cancer Cells

**DOI:** 10.1016/j.mcpro.2025.100944

**Published:** 2025-03-13

**Authors:** Yoon-Jin Lee, Chul Won Seo, Shinwon Chae, Chang Yeol Lee, Sang Soo Kim, Yoon-Hee Shin, Hyun-Mee Park, Yong Song Gho, Seongho Ryu, Sang-Han Lee, Dongsic Choi

**Affiliations:** 1 Department of Biochemistry, Soonchunhyang University, College of Medicine, Cheonan, Republic of Korea; 2 Department of Life Sciences, POSTECH, Pohang, Republic of Korea; 3 Advanced Analysis and Data Center, Korea Institute of Science and Technology, Seoul, Republic of Korea; 4 Soonchunhyang Institute of Medi-Bio Science (SIMS), Soonchunhyang University, Cheonan, Republic of Korea

**Keywords:** exosomes, extracellular vesicles, proteomics, cancer, metabolism

## Abstract

Most cancer cells adopt a less efficient metabolic process of aerobic glycolysis with high level of glucose uptake followed by lactic acid production, known as the Warburg effect. This phenotypic transition enables cancer cells to achieve increased cellular survival and proliferation in a harsh low-oxygen tumor microenvironment. Also, the resulting acidic microenvironment causes inactivation of the immune system such as T-cell impairment that favors escape by immune surveillance. While lots of studies have revealed that tumor-derived EVs can deliver parental materials to adjacent cells and contribute to oncogenic reprogramming, their functionality in energy metabolism is not well addressed. In this study, we established prostate cancer cells PC-3AcT resistant to cellular death in an acidic culture medium driven by lactic acid. Quantitative proteomics between EVs derived from PC-3 and PC-3AcT cells identified 935 confident EV proteins. According to cellular adaptation to lactic acidosis, we revealed 159 regulated EV proteins related to energy metabolism, cellular shape, and extracellular matrix. These EVs contained a high abundance of glycolytic enzymes. In particular, PC-3AcT EVs were enriched with apolipoproteins including apolipoprotein B-100 (APOB). APOB on PC-3AcT EVs could facilitate their endocytic uptake depending on low density lipoprotein receptor of recipient PC-3 cells, encouraging increases of cellular proliferation and survival in acidic culture media *via* increased activity and expression of hexokinases and phosphofructokinase. The activation of recipient PC-3 cells can increase glucose consumption and ATP generation, representing an acquired metabolic reprogramming into the Warburg phenotype. Our study first revealed that EVs derived from prostate cancer cells could contribute to energy metabolic reprogramming and that the acquired metabolic phenotypic transition of recipient cells could favor cellular survival in tumor microenvironment.

## Highlights

• Prostate cancer cell PC-3AcT was resistant to cellular death in an acidic medium.
• EVs from PC-3AcT cells were enriched with glycolytic enzymes.
• APOB in PC-3AcT EVs facilitated the endocytic uptake by LDLR of recipient cells.
• PC-3AcT EVs promoted the survival of PC-3 cells in an acidic medium by HK and PFKP.
• Reprogrammed PC-3 cells showed the Warburg phenotype depending on glycolysis.

## Graphical Abstract

**Figure:**
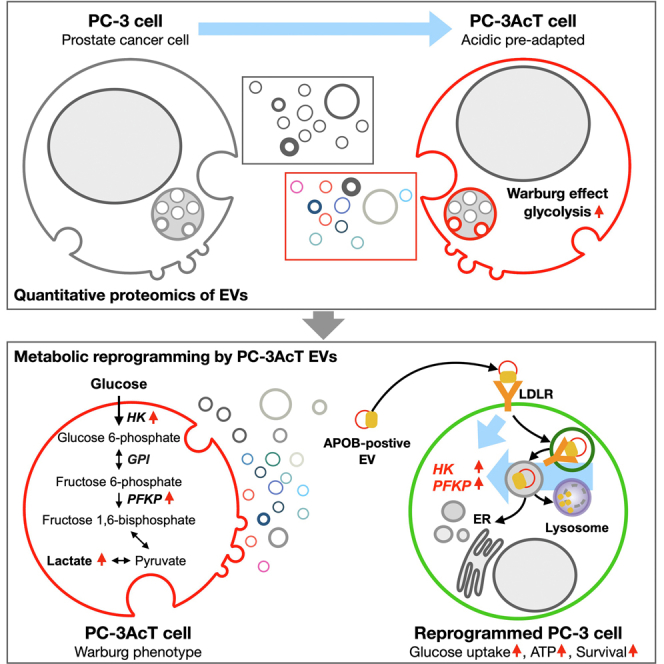

## In Brief

Quantitative proteomics between EVs derived from PC-3 and acidic pre-adapted isogenic counterpart PC-3AcT cells revealed regulated EV proteins related to energy metabolism. Also, these EVs contained abundant glycolytic enzymes. Particularly, PC-3AcT EVs were enriched with APOB. APOB-positive EVs could facilitate the endocytic uptake depending on the LDLR of recipient cells, encouraging increases of cellular proliferation and survival in acidic microenvironment *via* activation of HK and PFKP. EV-mediated reprogrammed PC-3 cells can increase glucose consumption and ATP generation, acquiring Warburg phenotype.

In tumor microenvironment, cancer cells are exposed to a low oxygen concentration with an abnormal vascular nature, in which they can adjust themselves to exhibit dynamic metabolic plasticity (1). Especially, they show fast proliferation adopting a less efficient metabolic process of anaerobic glycolysis with a higher level of glucose uptake and faster ATP generation than tricarboxylic acid (TCA) cycle–mediated oxidative phosphorylation (OXPHOS) (2), known as the Warburg effect (3). In this phenotypic transition about energy metabolism, ATP and other metabolic precursors can be rapidly produced, accelerating other metabolic pathways to generate amino acids, nucleotides, and lipids for cancer cell proliferation (4). This phenotypic transition enables cancer cells to achieve increased cellular survival with increased growth in a harsh low-oxygen tumor microenvironment (3). Enhanced glycolysis with decreased TCA cycle and OXPHOS enables faster generation of ATP with high production of lactate in both anaerobic and aerobic conditions (3, 5). Excess lactates are cotransported with protons into the extracellular space to prompt an extracellular acidic pH (6). Subsequent acidosis in the tumor microenvironment is closely related to oncogenic processes, including drug resistance (6) and inactivation of immune system such as T-cell impairment favoring the escape by immune surveillance (7).

Recently, extracellular vesicles (EVs) have been considered as major signaling mediators in the tumor microenvironment (8). EVs, an umbrella term, cover all types of secreted vesicles with sizes ranging from 30 nm to 1000 nm (9). They are composed of exosomes from intraluminal vesicles in multivesicular bodies, ectosomes shed from plasma membrane, EVs released from apoptotic cells, and other lipid bilayer particles (8). Importantly, EVs are equipped with diverse kinds of proteins, mRNA, miRNA, DNA, and bioactive lipids. They can transfer them to recipient cells (10). Accumulated evidence suggests that cancer cells actively release EVs into the tumor microenvironment to affect surrounding cells for tumor growth, invasion, metastasis, and escape from immune surveillance as well as neovascularization of adjacent endothelial cells (11). Recent studies have shown that their molecular components could be transferred to surrounding cells and influence their energy metabolism favoring cancer progression (12). Importantly, these metabolic transition of recipient cells could be stimulated by cargo transfer of EVs including metabolic enzymes such as PKM2, ALDOA, and ALDH3A1 by prompting glycolysis (13, 14) or other energy metabolism (12). These EV-mediated metabolic reprogramming of surrounding cells could influence their energy usage to favor cancer survival and progression in harsh tumor microenvironment. Although the functionality of EVs in metabolic remodeling of recipient cells has been reported, regulating vesicular proteome from cancer cells and their cargo transferring mechanism remain elusive.

In previous studies (15, 16, 17), we have established a prostate cancer cell line PC-3AcT that is resistant to cellular death in an acidic culture medium inducted by lactic acid, representing an increase of cellular survival by active glycolysis for ATP generation. Increased lactic acid level in prostate cancer is closely associated with cancer progression (18). Normal prostate cells mainly generate ATP by glucose oxidation from the TCA cycle for OXPHOS under aerobic conditions. However, advanced prostate cancer cells exhibit glycolysis-dependent Warburg phenotype, resulting in increased production of lactate (19, 20). High level of lactate in prostate cancer cells is related to disease recurrence, drug resistance, and a poor prognosis for survival (21, 22).

In this study, we applied quantitative proteomics to analyze EVs derived from parental PC-3 prostate cancer cells and their acidic pre-adapted isogenic counterparts (PC-3AcT). High purity EVs were isolated by combining size-exclusion chromatography (SEC) with high salt washing and ultracentrifuge as established in previous studies (23, 24). Isolated EVs were analyzed with a UHPLC-Orbitrap Eclipse Tribrid mass spectrometer using three biological replicates to reveal changes in EV-associated proteins from reprogrammed prostate cancer cells to the Warburg phenotype. Using a label-free quantitative proteomics, we identified a total of 935 EV proteins from both cell lines, including 159 proteins significantly regulated by lactic acidosis. These altered EV proteins were closely associated with gene ontology (GO) terms such as cholesterol efflux, cell junction, and integrin binding. Moreover, we found that PC-3 and PC-3AcT cells secreted EVs containing high levels of glycolysis-related enzymes including aldolase, GAPDH, PGK, ENO1, PKM, and LDH. Importantly, these proteomic transition of EVs enabled increased uptake of PC-3AcT EVs to recipient parental PC-3 cells by interaction between vesicular apolipoprotein B-100 (APOB) and cellular low-density lipoprotein receptor (LDLR) that mediated endocytic uptake, stimulating metabolic reprogramming in recipient cells. In conclusion, our results indicate that acidic-adapted prostate cancer cells may influence their biological activities of EVs through their proteomic changes, enabling to normal metabolic prostate cancer cells to transform to the Warburg phenotype.

## Experimental Procedures

### Cell Lines, Standard Culture Conditions, and Cell Viability

PC-3 cells, a human prostate cancer cell line, were purchased from the American Type Culture Collection. PC-3 cells were grown in Dulbecco's modified Eagle's medium (Welgene) supplemented with 5% fetal bovine serum (FBS) (Welgene), 1% L-glutamine (Hyclone), and 1% penicillin-streptomycin (Hyclone). Acidic pre-adapted PC-3 cells termed as PC-3AcT cells were established by continuously exposing PC-3 cells to 3.8 μM lactic acid with four serial passages during 15 days (15). PC-3AcT cells were maintained in normal culture media with 3.8 μM lactic acid. Cell viability was measured by MTT assay as previously described (16).

### Isolation of EVs

Cells were seeded into 150 mm plates at 4 × 10^6^ cells per 22 ml in Dulbecco's modified Eagle's medium containing 5% EV-depleted FBS with or without 3.8 μM of lactic acid for PC-3AcT and PC-3 cells, respectively. EV-depleted FBS was the supernatant of FBS after ultracentrifugation at 150,000*g* for 18 h following 0.22 μm filtration. After 3 days culture, conditioned media (CM) were collected and cell debris was removed by centrifugation at 400*g* for 10 min. The supernatant was further filtered through a bottle top filter with 0.22 μm pore size (GVS). Filtered CM was concentrated using an Amicon Ultra-15 centrifugal filter unit with 100,000 NMWL molecular cut-off (EMD Millipore). For the functional assay, one volume of concentrated CM was diluted with nine volumes of PBS and centrifuged at 110,000*g* for 70 min. Sedimented EVs were resuspended with PBS. For proteomic analyses, 500 μl of concentrated CM was loaded into a qEVoriginal SEC column (Izon Science). Then 2.5 ml of PBS was added. Flow-through was removed and EV-enriched fractions were collected following the addition of 1.5 ml of PBS (23). EV-enriched fraction was mixed with 1.5 ml of 2 M KCl in PBS and incubated for 30 min at 4 °C (23). EVs were sedimented by ultracentrifugation at 110,000*g* for 70 min and resuspended in 100 μl of lysis buffer containing 50 mM Tris–HCl (pH 7.5), 1% NP-40, 0.25% Na-deoxycholate, 100 mM NaCl, 1 mM EDTA, and protease inhibitor cocktail (Roche). Concentrations of proteins were quantified with a micro BCA assay (Thermo Fisher Scientific). To label EVs with fluorescent dye, concentrated CM was incubated with 10 μM of CellTrace Far Red (Thermo Fisher Scientific), 50 μM of DiO (Thermo Fisher Scientific), or 50 μM of DiR (Thermo Fisher Scientific) for 2 h at, room temperature in the dark. After 100 μl of concentrated CM with dye was loaded into the qEVsingle SEC column (Izon Science), 900 μl of PBS was added. Flow-through was removed and EV-enriched fractions were collected following the addition of 600 μl of PBS (23). All EVs were stored at −80 °C before use.

### Nanoparticle Tracking Analyses

The concentration and size of EVs were measured by nanoparticle tracking analysis (NTA). The NTA was performed at 25 °C for 30-s using a NanoSight NS300 instrument (Malvern) containing a 532 nm laser and the NTA software (version 3.0) three times. The camera level was set to 15 and the detection threshold was set to 10 for size and quantitation analyses.

### Western Blotting

Cell lysate was obtained using a cell lysis buffer containing 50 mM Tris–HCl (pH 7.5), 1% NP-40, 0.25% Na-deoxycholate, 100 mM NaCl, 1 mM EDTA, and protease inhibitor cocktail (Roche). For Western blotting, cells or EV proteins were mixed with a NuPAGE LDS sample buffer (Thermo Fisher Scientific) and 2.5% of 2-mercaptoethanol. Proteins were loaded into a NuPAGE 4 to 12% Bis-Tris gel (Thermo Fisher Scientific) and then transferred to a polyvinylidene difluoride membrane (GE Healthcare). The membrane was blocked with 5% nonfat milk in Tris-buffered saline containing 0.1% Tween 20 (TBST) for 2 h and incubated with indicated primary antibody at 4 °C overnight. The membrane was washed three times with 0.1% TBST for 10 min followed by incubation with a secondary antibody conjugated with horseradish peroxidase. Membrane detection was conducted using ECL Prime (Cytiva) and an X-ray film (Agfa). The following primary antibodies were used: rabbit anti-CD81 (dilution 1:1000, 56039S), rabbit anti-hexokinase 1 (HK1) (1:500, 2024S), rabbit anti-hexokinase 2 (HK2) (1:500, 2887), rabbit anti-phosphofructokinase (PFKP) (1:500, 8164), rabbit anti-p-ERK (1:500, 9101), rabbit anti-ERK (1:500, 9102S), rabbit anti-cyclin B1 (1:500, 12,231), rabbit anti-p-cdc42 (Thr161) (1:500, 9114S), rabbit anti-p-cdc2 (Tyr15) (1:500, 4539), mouse anti-β-actin (1:1000, 3700S) purchased from Cell Signaling Technology; mouse anti-CD9 (1:1000, MAB2529), mouse anti-fibronectin (FN1) (1:500, MAB1918) purchased from R&D Systems; mouse anti-cytochrome c (CYCS) (1:1000, 556,433) purchased from BD; rabbit anti-syntenin-1 (1:1000, ab19903), mouse anti-TSG101 (1:1000, ab125011) purchased from Abcam; mouse anti-β-catenin (CTNNB1) (1:500, sc-7963), rabbit anti-cyclin D1 (1:500, sc-718), mouse anti-pyruvate kinase M1/2 (PKM) (1:500, sc-365684) purchased from Santa Cruz Biotechnology; and OXPHOS Human WB antibody Cocktail (mouse anti-ATP5A, -UQCRC2, -SDHB, -COX2, -NDUFB8) (1:500, 45-8199) purchased from Invitrogen. The following horseradish peroxidase–conjugated secondary antibodies were used: goat anti-rabbit (1:5000, LF-SA8002) purchased from AB Frontier; goat anti-rabbit IgG (1:5000, AB205718) purchased from Abcam; and horse anti-mouse IgG (1:5000, 7076s) purchased from Cell Signaling Technology.

### Transmission Electron Microscope

EVs were fixed in 2% glutaraldehyde for 2 h at room temperature. Samples were divided into 3 μl and placed on parafilm. Carbon-coated grids (TED PELLA) were turned over and placed on the sample followed by 20 min of incubation. These grids were washed three times with drops of deionized water for 10-s and expanded to 2 μl of 1% uranyl acetate for negative staining. TEM was conducted after overnight drying samples at 4 °C. Transmission electron microscopy images were obtained with an electron microscope JEM1011 (JEOL) at an accelerating voltage of 80 kV.

### Metabolic Assays

PC-3 cells were seeded into a 96-well plate at a density of 1.5 × 10^4^ cells/well and incubated for 24 h. PC-3AcT EVs were used to treat PC-3 cells at 1.0 × 10^9^ particles in 200 μl of normal culture media at 5.0 × 10^9^ particles/ml. After 1 day, growth media were removed and PC-3AcT EVs were used to treat PC-3 cells again at the same concentration for 2 days. HK activity was measured spectrophotometrically according to instructions provided by the Hexokinase Colorimetric Assay Kit (BioVision). Glucose consumption was determined by assessing glucose content in the culture media according to the instructions provided by the Glucose Colorimetric Assay Kit (BioVision). Intracellular ATP content was determined by luminescence measurement using a CellTiter-Glo Luminescent Cell Viability Assay Kit (Promega) according to the manufacturer's instructions. Absorbance and luminescence values were measured with a Synergy HTX multi-mode reader (Biotek).

### Experimental Design and Statistical Analysis for Quantitative Proteomics of EVs

For proteomics analysis, EVs derived from PC-3 and PC-3AcT cells were analyzed with three independent biological replicates. A total 15 μg of EV proteins were subjected to in-gel digestion by trypsin as described in a previous study (25). Tryptic peptides were analyzed by LC-MS/MS with an Orbitrap Eclipse Tribrid mass spectrometer combined with an Ultimate 3000 UHPLC system (Thermo Fisher Scientific). Total peptides were dissolved in 20 μl of buffer A (0.1% formic acid in water) and 5 μl was loaded onto a trap column, Acclaim PepMap C18 nano Viper 100 (75 μm × 2 cm, 3 μm) (Thermo Fisher Scientific), at a flow rate of 5 μl/min with 95% buffer A for 4 min. These peptides were separated on an analytical column, PepMap RSLC C18 ES803A (75 μm × 50 cm, 2 μm) (Thermo Fisher Scientific) for 180 min with a gradient from 5% to 90% of solvent B (0.1% formic acid in acetonitrile) at a flow rate of 300 nl/min. The mass spectrometer was operated in a data-dependent top 20 scan mode switching between MS and MS2. Peptides were analyzed with the following parameters: 10 ppm for mass accuracy, 1850 V for ion spray voltage, 275 °C for the capillary temperature, m/z 375-1575 for the resolution of full scans, and 120,000 higher-energy collisional dissociation activation scans with 35% normalized collision energy. The quadrupole isolation window was 1.4 Da. MS/MS spectra were detected on the Orbitrap with a resolution of 30,000.

The raw file was analyzed using Maxquant software (version 2.2.0.0). UniProt human protein database (reviewed, released on 2022-08-29, 20,422 entries), a public protein database, was used with a fragment ion mass tolerance of 20 ppm and a parent ion tolerance of 20 ppm. The following modifications were applied: fixed modification for carbamidomethylation of cysteine (58 Da) and variable modification for oxidation of methionine (16 Da), deamidation of asparagine and glutamine (1 Da), and N-terminal acetylation (42 Da). Permission of two potential missed cleavages was applied for trypsin digestion. Database search results were further analyzed with Scaffold 5 software (version 5.2.2) (www.proteomesoftware.com/products/scaffold-5) using protein threshold ≥0.99% and peptide threshold ≥0.99% (peptide false discovery rate (FDR): 0.1%, protein FDR: 0.5%). Protein and peptide FDR were determined by Scaffold 5 using the probabilistic method employed by the Trans proteomic pipeline (26). These selected proteins were quantified by label-free protein quantitation integrated into Scaffold 5, employing the following options: statistical test (*t* test, no multiple test correction; *p*-value <0.1); fold change by category (PC-3 EVs as reference); use normalization, minimum value (0.0), and quantitative method (Weighted Spectra). Proteins with *p*-values less than 0.1 were considered significantly changed. Multiple test corrections using the Benjamini–Hochberg and Bonferroni methods in Scaffold 5 did not identify significantly changed proteins. Alternatively, we calculated the *p*-values using Student's *t* test without multiple test correction in Scaffold 5, where the confidence of label-free quantitation was validated in the previous studies (23, 24, 27). The mass spectrometry proteomics data have been deposited to the ProteomeXchange Consortium *via* the PRIDE partner repository with the dataset identifier PXD054467.

### Experimental Design and Statistical Analysis for Transcriptomics of PC-3, PC-3AcT, and PC-3AcT EV-Treated PC-3 Cells

For transcriptomics analysis, PC-3, PC-3AcT, and PC-3AcT EV-treated PC-3 cells were analyzed with three independent biological replicates. Total RNA from PC-3, PC-3AcT, and PC-3 treated with PC-3AcT EVs at 1 × 10^7^ cells were extracted using Trizol (Thermo Fisher Scientific) according to the manufacturer's instructions. Total RNA concentration was calculated by Quant-iT RiboGreen (Invitrogen). To assess the integrity of the total RNA, samples are run on the TapeStation RNA ScreenTape (Agilent Technologies). Only high-quality RNA preparations with RNA integrity number greater than 7.0 were used for RNA library construction. A library was independently prepared with 1 μg of total RNA for each sample by Illumina TruSeq Stranded mRNA Sample Prep Kit (Illumina, Inc) by the manufacturer's instructions. The libraries were quantified using KAPA library quantification kits for Illumina sequencing platforms according to the qPCR quantification protocol guide (KAPA Biosystems) and qualified using the TapeStation D1000 ScreenTape (Agilent Technologies). Indexed libraries were submitted to an Illumina NovaSeqX (Illumina) at Macrogen and the paired-end (2 × 100 bp) sequencing was performed. Paired-end sequencing reads were generated on the Illumina sequencing NovaSeqX platform (Illumina). Before starting the analysis, Trimmomatic v0.38 was used to remove adapter sequences and trim bases with poor base quality. The cleaned reads were aligned to the *Homo sapiens* (GRCh38) using HISAT v2.1.0 (28). The reference genome sequence and gene annotation data were downloaded from the NCBI Genome assembly and NCBI RefSeq database, respectively. Aligned data (SAM file format) were sorted and indexed using SAMtools (v1.9). After alignment, the transcripts were assembled and quantified using StringTie (v2.1.3 b) (29). Gene-level and transcript-level quantification were calculated as raw read count, FPKM (fragments per kilobase of transcript per million mapped reads), and TPM (transcripts per million). Statistical analyses of differential gene expression were performed by DESeq2 (v1.38.3) using raw counts as input (30). Genes with nonzero counts in all samples were selected. A filtered data set was applied with relative log expression normalization to correct the variation of library sizes among samples. The statistical significance of the differential expression gene was determined using DESeq2 nbinom WaldTest (30). Fold change and *p*-value were extracted from the result of WaldTest. All *p*-values are adjusted by the Benjamini–Hochberg algorithm to control the FDR. The significant gene list was filtered by fold change ≥2 and raw *p*-value <0.05. The datasets were submitted to Gene Expression Omnibus and under submission number GSE283647.

### Variant Calling Analysis for SNPs

For variant calling of RNA-Seq data, trimmed reads were aligned to *H. sapiens* (GRCh38) with STAR (31) and then duplications were marked and discarded using SAMBAMBA markdup (broadinstitute.github.io/picard/). Afterward, aligned reads that can be used in analysis are created through Split “N” Trim and base recalibration process. The aligned reads are used for variant calling with HaplotypeCaller module of GATK v4.2.0.0 (32). Variant filtering for each sample was performed base on Fisher strand values (FS > 30.0) and Qual By Depth values (QD < 2.0) in the VariantFiltration module of GATK and additionally filtered out variants with depth of coverage (DP < 10) using an in-house script.

### GO and Protein Interaction Network Analyses

Lists of identified proteins were imported into the DAVID Bioinformatics database (davidbioinformatics.nih.gov) and assigned to their GO annotations as biological process, cellular component, and molecular function. Information of protein–protein interactions was acquired from the BioGRID database (version 4.4.229; http://www.thebiogrid.org/). The network for identified EV proteins was constructed using Cytoscape (www.cytoscape.org/).

### Fluorescent Microscope for EV Uptake

A total 10,000 of PC-3 cells were seeded into *μ*-Slide 8-Well ibiTreat chambered coverslips (ibidi) and cultured for 24 h. To compare the uptake efficiency between PC-3 and PC-3AcT EVs in PC-3 cells, CellTrace Far Red–labeled PC-3 or PC-3AcT EVs were used to treat PC-3 cells at 2 × 10^9^ particles/ml for 18 h. For dynasore (Selleckchem) treatment for PC-3AcT EV uptake efficiency, growth media of PC-3 cells were replaced with cell culture media containing 50 μM dynasore and incubated for 1 h. DiR-labeled PC-3AcT EVs were added to PC-3 cells at 1 × 10^9^ particles/ml and incubated for 3 h or 7 h. For anti-LDLR (R&D Systems) treatment to determine PC-3AcT EV uptake efficiency, growth media of PC-3 cells were replaced with cell culture media containing anti-LDLR (3 μg/ml) and incubated for 1 h. DiO-labeled PC-3AcT EVs were added to PC-3 cells at 1 × 10^9^ particles/ml and incubated for 7 h. For lysosomal trafficking of taken PC-3AcT EVs, DiR-labeled PC-3AcT EVs were added to PC-3 cells at 1 × 10^9^ particles/ml and incubated for 7 h. After EV incubation, growth media of PC-3 cells were removed and cells were incubated with 500 nM endoplasmic reticulum (ER)-Tracker (Thermo Fisher Scientific) for 30 min or 200 nM LysoTracker for 2 h (Thermo Fisher Scientific) in culture media. For fluorescence images, cells were fixed with 4% paraformaldehyde. Images were acquired and analyzed using an Agilent Lionheart FX Automated Microscope (BioTek).

## Results

### Isolation and Characterization of EVs Between Prostate Cancer PC-3 and Acidic Pre-adapted PC-3AcT Cells

**Fig. 1:**
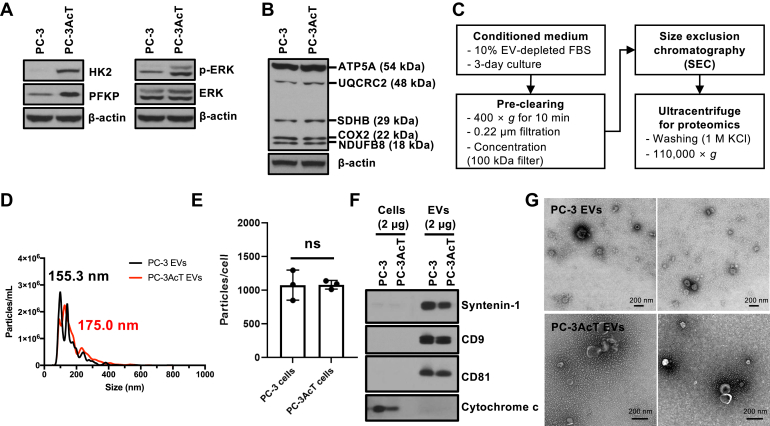
**Isolation and characterization of PC-3 and PC-3AcTEVs.***A*, Western blotting showing increased expression of HK2 and PFKP with activated ERK signaling pathway (HK2 and PFKP are rate-limiting enzymes in glycolysis). *B*, expression levels of mitochondrial OXPHOS proteins not changed in PC-3AcT cells. *C*, diagram showing a schematic workflow of the isolation of EVs for proteomics. *D* and *E*, NTA showing size and released number of EVs in PC-3 and PC-3AcT EVs. *F*, Western blotting showing relative enrichment of canonical EV marker proteins such as Syntenin-1, CD9, and CD81 but depletion of non-EV protein cytochrome c. Western blotting analyses were conducted using independent biological replicate of isolated EVs, distinct from those used in proteomics. *G*, TEM images showing a spherical round shape of PC-3 and PC-3AcT EVs consistent with sizes measured by NTA.

In previous research studies (15, 16, 17), acidic pre-adapted PC-3AcT cells showed significantly increased cellular proliferation in an acidic microenvironment induced by lactic acid than their parental PC-3 cells, representing a highly tolerant phenotype of PC-3AcT cells in acidic microenvironment caused by activation of glycolysis. Western blotting showed increased cellular expression of HK2 and PFKP known to be key regulatory enzymes involved in rate-limiting and committed steps of glycolysis and increased phosphorylation of ERK1/2 signaling pathway for the cellular growth (Fig. 1*A*). On the other hand, mitochondrial electron transport chain in OXPHOS including ATP synthase subunit alpha mitochondrial (ATP5A), cytochrome b-c1 complex subunit 2 mitochondrial (UQCRC2), succinate dehydrogenase [ubiquinone] iron-sulfur subunit, mitochondrial (SDHB), cyclooxygenase-2 (COX II), and NADH dehydrogenase [ubiquinone] 1 beta subcomplex subunit 8 (NDUFB8) were not affected by lactic acid adaptation in PC-3AcT cells (Fig. 1*B*). These results indicate that the increased proliferation of PC-3AcT cells is closely related to the utilization of glucose and subsequent activation of glycolysis rather than the TCA cycle coupled with OXPHOS, indicating a Warburg-like metabolism in PC-3AcT cells as reported previously (33, 34). Transcriptomic analyses between PC-3 and PC-3AcT cells revealed differential expression patterns, with 776 downregulated and 186 upregulated mRNA transcripts, respectively (supplemental Fig. S1*A* and supplemental Table S1). GO and KEGG analyses of differentially regulated mRNA identified enriched annotations related to cell adhesion, signal transduction, plasma membrane, and calcium ion regulation (supplemental Fig. S1*B*). Furthermore, PC-3 and PC-3AcT cells contain SNPs in comparison to the reference sequence (supplemental Fig. S2*A* and supplemental Table S2). It is noteworthy that PC-3AcT is also further mutated, containing approximately 6000 of additional SNPs from parental PC-3 cells (supplemental Fig. S2*B*).

This cellular metabolic transition could be coupled with regulated release of EVs with changes in their molecular contents (12). To understand the impact of vesiculation on PC-3AcT cells in an acidic microenvironment, EVs were isolated with previously established procedure (23) (Fig. 1*C*). Cells were cultured in culture media containing 10% EV-depleted FBS for 3 days. CM was precleared by centrifugation at 400*g*, further filtered with a 0.22 μm pore-sized membrane filter, and concentrated with a 100-kDa centrifugal filter. Crude EVs were isolated by SEC. Size and concentration of these EVs were measured by NTA (average size: 155.5 nm and 175.0 nm for PC-3 EVs and PC-3AcT EVs, respectively) (Fig. 1*D*). Average size of PC-3AcT EVs was slightly bigger than that of PC-3 EVs. However, the difference between the two was not significant. Total released EV number per cell was similar between PC-3 and PC-3AcT cells (Fig. 1*E*). For proteomics, crude EVs by SEC were further washed with 1 M KCl to remove nonspecifically bound proteins on EVs as described in a previous study (23). Western blotting showed enrichment of canonical EV marker proteins including syntenin-1 and CD81. However, cytochrome C was depleted in isolated PC-3 and PC-3AcT EVs (Fig. 1*F*). TEM showed a canonical round cup-shaped morphology of EVs (Fig. 1*H*), implying that our EVs were confidently isolated.

### Global Proteomes of PC-3 and PC-3AcT Cell-Derived EVs

**Fig. 2:**
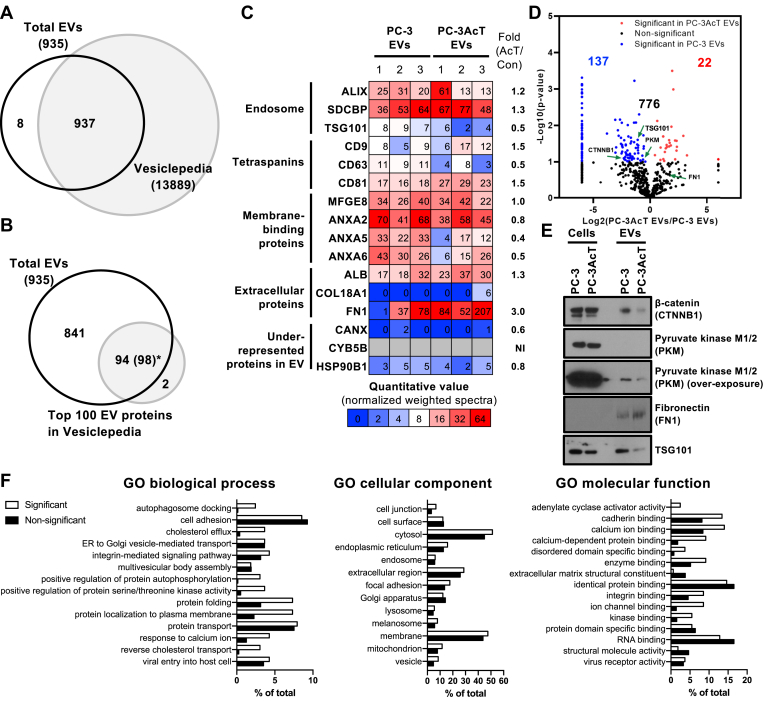
**Quantitative proteomics of PC-3 and PC-3AcT EVs.***A*, comparison of identified EV proteins with Vesiclepedia (www.microvesicles.org). Venn diagram indicated that the majority of both EV proteins were previously known EV proteins. *B*, most of top 100 EV proteins frequently identified in Vesiclepedia are included in our EV proteome. *C*, heatmap showing enrichment of EV marker proteins related to endosome, tetraspanins, membrane-binding proteins, and EV-associated proteins, which are canonical EV proteins by MISEV guidelines (36). *D*, differentially regulated proteins in PC-3 and PC-3AcT EVs are indicated in a volcano plot. Representative proteins validated by Western blotting are indicated by a *green* arrow. *E*, Western blotting showing differential expression of proteins including CTNNB1, PKM, FN1, and TSG101 as quantified in proteomics. Additionally, CTNNB1 was further validated in independently isolated EVs to confirm its differential expression observed in quantitative proteomics (supplemental Fig. S4). Western blotting analyses were performed using independent biological replicate of isolated EVs, separate from those used in proteomics. *F*, GO analyses for biological process, cellular component, and molecular function in significant and nonsignificant regulated proteins between PC-3 and PC-3AcT EVs (*p*-value <0.1).

To address detailed proteomic changes for EVs affected by acidic tolerance of PC-3AcT cells, we applied mass spectrometry–based proteomics. A total of 15 μg of EVs proteins were analyzed by in-gel digestion and LC-MS/MS with three biological replicates. Raw MS files were analyzed by MaxQuant with UniProt-reviewed human proteome database. Confident proteomes were defined by Scaffold with statistical confidence at protein threshold ≥99.0% and peptide threshold ≥99.0%. Finally, a total of 935 proteins were identified in PC-3 and PC-3AcT EVs (supplemental Tables S3–S5). These EV proteins were compared with proteins listed in the Vesiclepedia database (www.microvesicles.org), a web-based database of known EV components (35) (supplemental Tables S3–S5). As expected, the majority of proteins identified in proteomes were previously known to be EV-associated proteins in Vesiclepedia (Fig. 2*A* and supplemental Fig. S3*A*). Notably, only eight proteins in our list were previously unreported EV cargo proteins. These proteins were detected at low spectral counts from one to three of weighted spectra (supplemental Fig. S3*A* and supplemental Table S3). Moreover, we compared our EV proteomes with the top 100 proteins frequently identified in EV studies defined by Vesiclepedia (35). A total of 98 proteins of the top 100 EV proteins were identified in our EV proteomes with the exception of HIST1H4A and adenylyl cyclase–associated protein 1. Collectively, these results represent confident identification of proteins in EV proteomes of this study.

We further checked the abundance of canonical EV marker proteins as suggested by MISEV (36). EV proteins were quantified by label-free quantitation with normalized weighted spectra in Scaffold 5 program (23). As expected, higher quantitation values were observed for proteins related to endosome (ALIX, syntenin-1), tetraspanins (CD9, CD63, CD81), membrane-binding proteins (MFGE8, annexins), and proteins often associated with EV surfaces (albumin and extracellular matrix fibronectin). Consistent with this pattern, we observed lower abundance or absence of proteins that normally tended to be excluded from the EV cargo (such as CANX (calnexin), CYB5B (cytochrome B5 type B), and HSP90B1, also known as Grp94 located in the cytoplasmic ER, mitochondria, and Golgi apparatus, respectively) in the EV proteome (36, 37). Impressively, among EV marker proteins, FN1 had a higher expression level at about 3-fold whereas ANXA5 and ANXA6 had lower expression levels at about 0.4- and 0.5-fold in PC-3AcT EVs, respectively. These proteins could be located on the EV surface as EV corona proteins. They might be related to the uptake of EVs by cells (24, 38). Collectively, our EV isolations and proteomic analyses confidently identified expected marker proteins and found differentially regulated proteins on PC-3AcT EVs.

### The Impact on EV Proteome is Affected by Acidic Tolerance of PC-3AcT Cells

We conducted the quantitative proteomic analyses as described in our previous research, which validated the quantitative values obtained through label-free quantitation by Western blotting. (23, 24, 27). To discriminate substantial alterations in proteins between PC-3 and PC-3AcT EVs, regulated EVs were categorized based on statistical significance, with *p*-values less than 0.01, 0.05, and 0.1, resulting in the identification of 40, 111, and 159 significant proteins, respectively (Fig. 2*D* and supplemental Fig. S3*B*). Representative proteins, including CTNNB1 (*p*-value = 0.0800), PKM (*p*-value = 0.0870), FN1 (*p*-value = 0.220), and TSG101 (*p*-value = 0.025) were validated by Western blotting (Fig. 2*E* and supplemental Fig. S4). It is noteworthy that proteins with *p*-values less than 0.1 were confidently confirmed to have differential expression in both label-free quantitation and Western blotting. Consequently, we categorized significant regulated EV proteins with *p*-values less than 0.1. As a result, a total of 137 proteins and 22 proteins were significantly upregulated in PC-3 EVs and PC-3AcT EVs, respectively (Fig. 2*D*). To find possible clues to the functional role of EV-associated proteins, we analyzed our proteomes against GO terms for biological processes, cellular components, and molecular function in DAVID database (Fig. 2*F*). With respect to GO biological process analysis, we found that categories such as autophagosome docking, cholesterol efflux, phosphorylation, protein folding, and protein trafficking were significantly changed in PC-3AcT EVs. For GO cellular components, overall terms were similar between significant and nonsignificant EV proteomes. However, cell junction–related proteins were more affected in acidic tolerance PC-3AcT cells. Especially, CTNNB1 was downregulated in PC-3AcT EVs. This was validated by Western blotting (Fig. 2*E*). Also, lots of significant regulated proteins were categorized in protein binding–related terms such as cadherin binding, calcium ion binding, enzyme binding, and integrin binding of GO molecular function. These features of PC-3AcT EV proteome suggest a preferential selection of EV proteins related to their biogenesis, biological functions, and targeting involved in energy metabolism, cellular shape, and extracellular matrix (ECM) recognition in the tumor acidic microenvironment. It should be noted that the overall proteomic composition between EVs was relatively similar due to isogenic origin of PC-3 and PC-3AcT cells as shown in substantial contribution of common 776 (83.0%) proteins.

### PC-3 and PC-3AcT EVs Equipped with Abundant Glycolytic Enzymes

**Fig. 3:**
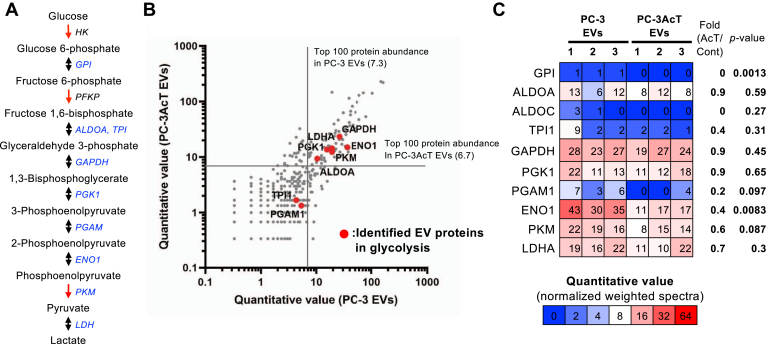
**Enrichment of glycolysis-related enzymes in PC-3 and PC-3AcT EVs.***A*, glycolysis pathway was indicated by the *blue letter* of identified EV proteins. *B*, a scatter plot showing quantitative values of proteins in PC-3 and PC-3AcT EVs. Identified glycolytic enzymes in EVs are indicated by a *red* color–filled circle. Note that proteins identified only in PC-3 and PC-3AcT EVs are not plotted. *C*, heatmap showing abundance enrichment of glycolysis-related enzymes in EVs.

Previous research studies have reported metabolic enzymes related to glycolysis, including PKM2, ALDOA, and ALDH3A1 carried by EVs that are closely related to the functionality of EVs to promote glycolytic metabolism of recipient cells (13, 14) and bone metastasis of prostate cancer cells (39). We investigated the expression of glycolytic enzymes in EVs. Most enzymes were identified in both EVs except HK and PFKP (Fig. 3*A*). Quantitative proteomic analyses indicated that GAPDH, PGK, ENO1, PKM, LDHA, and ALDOA were abundantly expressed in both PC-3 and PC-3AcT EVs (Fig. 3*B*). Among them, GAPDH and PGK amounts remained similar in both EVs. However, ENO1, PKM, and LDHA were relatively lower in PC-3AcT EVs than in PC-3 EVs (Fig. 3*C*), possibly suggesting that some glycolytic enzymes in EVs released from parental cells were regulated in the acidic microenvironment. These vesicular metabolic enzymes in EVs are linked to EV-mediated intercellular functionality in cancer progression and metastasis (40).

### PC-3AcT EVs Transform Parental PC-3 Cells to Acquire Acidic Resistance *via* Transition to the Warburg Phenotype

**Fig. 4:**
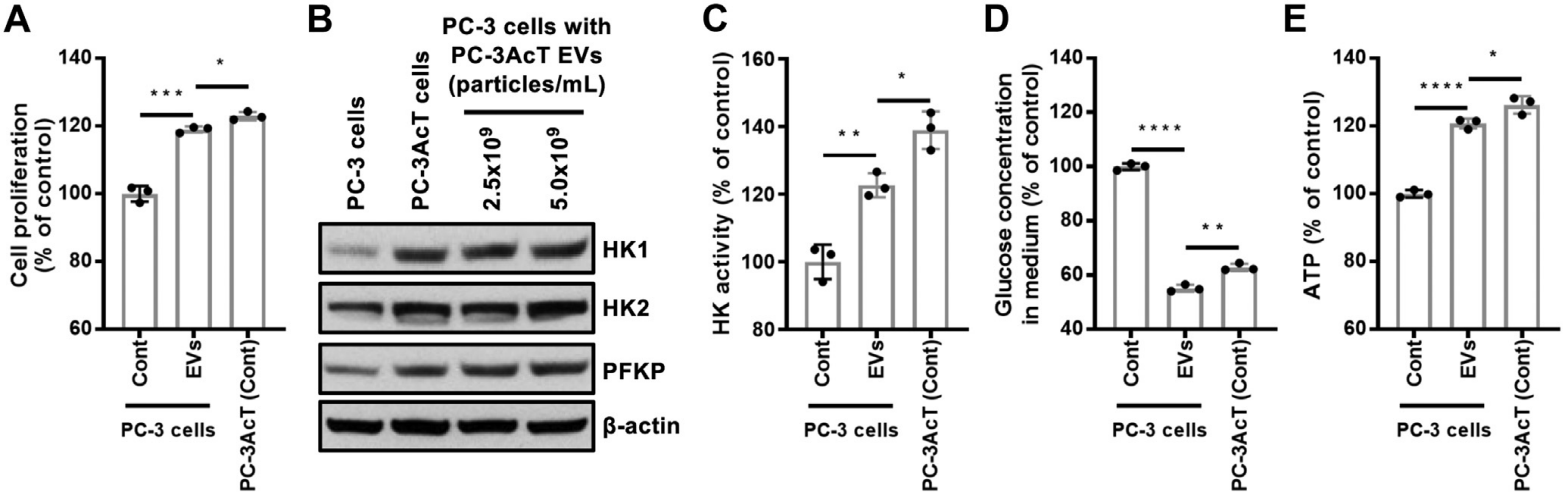
**Metabolic reprogramming of PC-3 cells treated by PC-3AcT EVs.***A*, MTT assay showing significantly increased cellular proliferation of PC-3 cells treated by PC-3AcT EVs in acidic culture media induced by lactic acid. Control is PC-3 cells without PC-3AcT EV treatment. *B*, HK1, HK2, and PFKP are overexpressed in reprogrammed PC-3 cells by PC-3AcT EVs. *C*–*E*, HK activity, glucose consumption, and ATP generation are significantly increased in reprogrammed PC-3 cells by PC-3AcT EVs. *p*-values (∗∗∗∗ < 0.0001; ∗∗∗ < 0.001, ∗∗ < 0.01, ∗ < 0.05).

To address metabolic reprogramming by PC-3AcT EVs, parental PC-3 cells were treated with PC-3AcT EVs at 5.0 × 10^9^ particles/ml once daily for 2 days. The MTT assay demonstrated a significant increase in the cellular proliferation of reprogrammed PC-3 cells by PC-3AcT EVs in acidic culture media induced by lactic acid (Fig. 4*A*). Notably, the cellular proliferation of reprogrammed PC-3 cells was approximately 118.0% compared to the average 122.9% of PC-3AcT cells. To reveal metabolic changes affected by PC-3AcT EVs, cellular expression levels of HK1, HK2, and PFKP known to be regulatory enzymes in rate-limiting steps of glycolysis were checked. Western blotting analyses showed the expression levels of these enzymes in reprogrammed PC-3 cells were upregulated by PC-3AcT EVs (Fig. 4*B*), favoring the activation of glycolysis. We further confirmed that the enzymatic activity of HK in reprogrammed PC-3 cells was significantly increased by PC-3AcT EV treatment, average 122.7%, while HK activity was significantly higher in PC-3AcT cells, average 139.0% (Fig. 4*C*). These increased expression and enzymatic activity resulted in increased consumption of glucose (Fig. 4*D*) and subsequent generation of high-level of ATP (Fig. 4*E*) in both PC-3 cells treated by PC-3AcT EVs and PC-3AcT cells. Collectively, PC-3 cells stimulated by PC-3AcT EVs were reprogrammed to the Warburg phenotype with increased glycolytic energy metabolism. In comparison between PC-3AcT EV-treated PC-3 cells and PC-3AcT cells, PC-3AcT cells exhibited significantly higher HK activity, accompanied by increased glucose consumption and ATP generation. Although the elevated HK activity, glucose consumption, and ATP generation in PC-3AcT cells, the resulting cell proliferation difference was not substantial. This implies that the multifaceted functionality of PC-3AcT EVs not only activated the expression of HK but also stimulated other signaling pathways for cell proliferation.

**Fig. 5:**
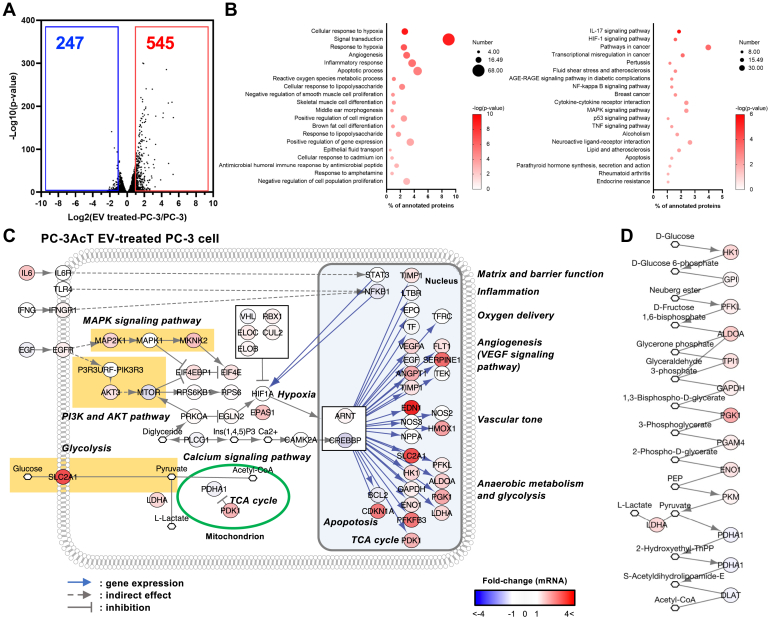
**Transcriptomics of PC-3 and PC-3 treated by PC-3AcT EVs (EV-treated PC-3).***A*, significantly altered mRNA were selected with a fold change greater than 2 and a *p*-value less than 0.05. In volcano plots, significantly downregulated mRNA was indicated with *blue* numbers, and upregulated mRNA with *red* numbers. *B*, a total of 792 significantly regulated proteins was analyzed by GO biological process and KEGG pathway analysis using the DAVID bioinformatics database (davidbioinformatics.nih.gov). *C*, differentially regulated EV proteins were mapped in KEGG HIF-1 signaling pathway annotation. *D*, glycolysis pathway–related mRNA is mapped based on their fold change.

To further evaluate the systemic transformation of PC-3 cells induced by PC-3AcT EVs, transcriptomic analyses were conducted between PC-3 and PC-3AcT EV-treated PC-3 cells (supplemental Table S1). A total of 247 and 545 mRNAs exhibited significant downregulation and upregulation with more than 2-fold in PC-3 cells and PC-3AcT EV-treated PC-3 cells, respectively (Fig. 5*A*). GO biological process and KEGG pathway analyses of differentially regulated 792 mRNA revealed significant enrichment of hypoxia-related annotations, including cellular response to hypoxia and response to hypoxia, and HIF-1 signaling pathway (Fig. 5*B*). Given the well-established role of HIF-1 as a key regulator of cancer metabolism and Warburg phenotype (41), we further mapped the mRNA in HIF-1 signaling pathway (Fig. 5*C*). Notably, PC-3AcT EV-treated PC-3 cells exhibited elevated expression of mRNA associated with the MAPK signaling pathway, which regulates cell proliferation, and the AKT signaling pathway, which promotes cellular survival. These overexpressions are consistent with our observations of increased cell proliferation in PC-3AcT EV-treated PC-3 cells (Fig. 4*A*). Furthermore, downstream proteins regulated by HIF1A and EPAS1, known as HIF2A, were also overexpressed, which are associated with angiogenesis, vascular tone, apoptosis, and glycolysis (Fig. 5*C*). This suggests the potent effect of PC-3AcT EVs on PC-3 cells for metabolic reprogramming. Remarkably, glycolysis-related mRNA exhibited significant upregulation. SLC2A1, known as GLUT1, was highly overexpressed, facilitating increased cellular uptake of glucose from extracellular space for glycolysis, consistent with the increased glucose consumption in PC-3AcT EV-treated PC-3 cells (Fig. 4*C*). However, the key activator PDHA1 (subunit of pyruvate dehydrogenase) in the TCA cycle was downregulated, while the negative regulator PDK1 (pyruvate dehydrogenase kinase 1) was overexpressed indicating that the TCA cycle is not activated by PC-3AcT EVs (Fig. 5*C*). Subsequently, we assessed the expression of mRNA related to glycolysis and TCA cycle. Most of the glycolysis-related mRNA exhibited increased expression in PC-3AcT EV-treated PC-3 cells, whereas TCA cycle–related mRNA remained unchanged or was downregulated (Fig. 5*D*). Furthermore, glycolysis-related genes are genetically mutated or regulated *in vivo* in prostate cancer tissues from the TCGA PanCancer Atlas (www.cbioportal.org), which provides data on structural variants, mutations, copy number variations, and mRNA expression of these genes in prostate cancer (supplemental Fig. S5). This *in vivo* relevance of glycolysis-related gene mutations highlights the systemic impact of PC-3AcT EVs on the metabolic reprogramming of recipient PC-3 cells, offering a novel perspective for understanding the prostate cancer microenvironment *in vivo*. Specifically, it suggests that the metabolic and molecular transformations mediated by EVs play a crucial role in cellular adaptation and tumor progression within the acidic tumor microenvironment.

### PC-3AcT EVs are Actively Taken up by PC-3 Cells with Increased ECM Proteins and Apolipoproteins

**Fig. 6:**
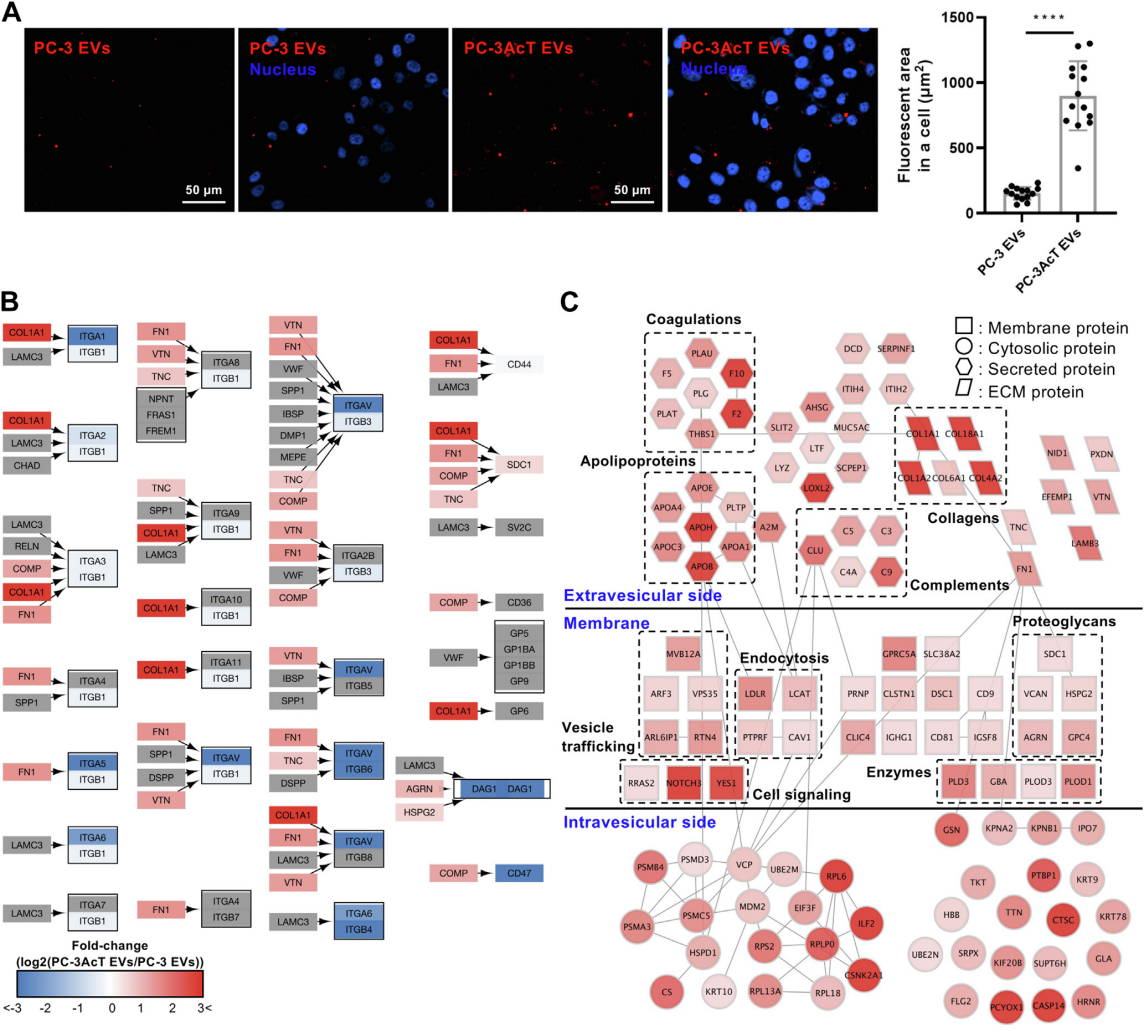
**Increased uptake of PC-3AcT EVs and regulated proteins related to their uptake by PC-3 cells.***A*, far *red*–labeled EVs at 2.0 × 10^9^ particles/ml were used to treat PC-3 cells and their taken fluorescent EVs were measured by fluorescence microscope. PC-3AcT EVs showed better uptake than PC-3 EVs in PC-3 cells. Please note that fluorescent spots that exceed 1 μm represent clusters of labeled EV-derived components or aggregates in cellular compartments (*e.g.*, endosomes or lysosomes). *B*, differentially regulated EV proteins were mapped in KEGG ECM–receptor interaction annotation. Collagen COLA1, FN1, and other ECMs were overexpressed in PC-3AcT EVs. *C*, protein–protein interaction network among 1.5-fold upregulated PC-3AcT EV proteins was mapped in the BioGRID protein interaction database and their interactions were visualized by Cytoscape (cytoscape.org). EV proteins were grouped based on their main functionality and related proteins as indicated by a *dashed line* box. *p*-values (∗∗∗∗ < 0.0001).

To further understand the changed functionality of PC-3AcT EVs affected by acidosis in tumor microenvironment, we analyzed the uptake efficiency between PC-3 and PC-3AcT EVs. EVs were labeled a CellTrace Far Red chemical dye permeant to EV membrane. Upon entering the intravesicular compartment, the dye undergoes cleavage of its acetate groups by intravesicular esterases, rendering it impermeable to reside within EVs. (8). Furthermore, the succinimidyl ester group of the dye can react with free amine groups of EV components, such as proteins, to generate covalent conjugates, thereby stabilizing the retention of the fluorescent signal within EVs (8). During the uptake of EVs, it can be trafficked or degraded while still retaining the CellTrace Far Red dye attached to their components. This allows for the tracking of the destination of the EV component in the recipient cells. Far red–labeled fluorescent EVs at 2.0 × 10^9^ particles/ml were used to treat PC-3 cells. Their taken (fluorescence) by EVs was measured with a fluorescence microscope (Fig. 6*A*). PC-3AcT EVs showed a greater uptake than PC-3 EVs in PC-3 cells (Fig. 6*A*), implying that their increased uptake was closely related to metabolic reprogramming of recipient PC-3 cells.

To glean possible clue to functional EV-associated proteins in preferential PC-3AcT EV uptake, we first analyzed surface integrin and ECM proteins possibly involved in EV uptake (24). KEGG pathway ECM–receptor interaction was mapped with identified proteins in PC-3AcT EVs using quantitative values (Fig. 6*B*). Impressively, integrin proteins were not overexpressed whereas COLA1 and FN were overexpressed on PC-3AcT EVs. It was notable that vesicular ECM as initial molecular recognition was implicated in bridging EV-cell contact prior to uptake, including the requirement for expressing heparin sulfate proteoglycan (42, 43) or for interacting with integrin receptors (44) by EV recipient cells.

Next, we evaluated the interaction network between 1.5-fold upregulated proteins in PC-3AcT EVs comparing PC-3 EVs. Among 146 upregulated proteins, 112 proteins were mapped in the BioGRID protein–protein interaction database and visualized by Cytoscape (Fig. 6*C*). We subdivided their subvesicular location in EVs based on UniProt annotation about subcellular location. The protein–protein interaction network points to a functional connection with apolipoproteins, coagulation, complements, and collagens with membrane proteins related in vesicle trafficking, endocytosis, proteoglycans, and cell signaling pathways. In this regard, APOB was significantly enriched in PC-3AcT EVs by 7.6-fold with an abundant quantitative value of 146.7. Curiously, APOB on low-density lipoprotein particles was recognized by LDLR for their uptake by receptor-mediated endocytosis, potentially indicating that this uptake was an applicable route in APOB-positive EVs. Although APOB is frequently identified in blood EVs in various studies (45, 46), its sorting during EV biogenesis and regulation during the acquisition of the Warburg phenotype, especially in prostate cancer, remain unknown.

### PC-3AcT EV Internalization Depends on APOB and LDLR

**Fig. 7:**
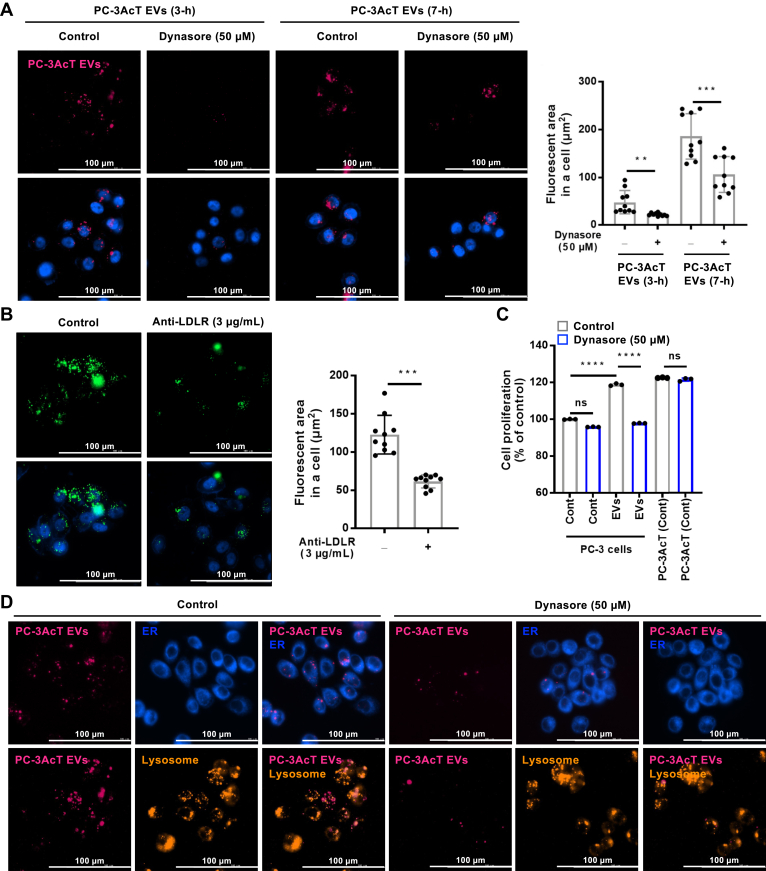
**Endocytic uptake of PC-3AcT EVs by vesicular APOB recognized by cellular LDLR.***A*, DiR-labeled EVs at 1.0 × 10^9^ particles/ml were used to treat PC-3 cells with or without dynasore, an endocytosis inhibitor, and fluorescent EVs taken were measured by fluorescence microscope. Increased PC-3AcT EV uptake in PC-3 cells was inhibited by dynasore treatment. *B*, anti-LDLR neutralizing antibody treatment to PC-3 cells inhibited the uptake of PC-3AcT EVs at a similar extent to that obtained in the presence of dynasore, implying that this endocytic uptake depended on vesicular APOB recognition by cellular LDLR. Please note that fluorescent spots that exceed 1 μm represent clusters of labeled EV-derived components or aggregates in cellular compartments (*e.g.*, endosomes or lysosomes). *C*, MTT assay showing that increased cellular proliferation of reprogrammed PC-3 cells by PC-3AcT EVs was inhibited by the presence of dynasore, representing that APOB-carrying EVs were functional regulators for energy metabolic reprogramming in recipient PC-3 cells. *D*, staining for fluorescent organelle trackers with taken DiR-labeled PC-3AcT EVs revealed that PC-3 cells could concentrate ingested EVs in the lysosome and to a lesser extent in the ER. *p*-values (∗∗∗∗ < 0.0001; ∗∗∗ < 0.001; ∗∗ < 0.01).

To assess the contribution of APOB to EV internalization by LDLR in recipient cells, we treated EVs with dynasore, a dynamin-dependent endocytosis inhibitor involved in receptor-mediated endocytosis. Inhibitor treatment markedly reduced the cellular uptake of PC-3AcT EVs after dynasore treatment (Fig. 7*A*). To address the total taken EVs from total treated PC-3AcT EVs, we measured residual fluorescent DiO-labeled PC-3AcT EVs in conditioned medium after 7 h of EV treatment. Approximately 45% of DiO-labeled EVs remained, indicating that around 55% of EVs were taken or processed by PC-3 cells (supplemental Fig. S6). Moreover, when EVs were incubated with target cells in the presence of an anti-LDLR neutralizing antibody, their uptake was impaired to an extent similar to that obtained in the presence of dynasore, implying that this endocytic uptake was dependent on vesicular APOB recognition to cellular LDLR (Fig. 7*B*). We further evaluated the functionality of APOB-carrying PC-3AcT EVs in the presence of dynasore (Fig. 7*C*). As expected, MTT assay showed cellular proliferation of PC-3 cells treated by PC-3AcT EVs in the presence of dynasore was evidently reduced, implying that APOB on PC-3AcT EVs was recognized by LDLR and that it facilitated their endocytosis in PC-3 cells. These results suggest that APOB present on EV surface contributes to elevated EV uptake by PC-3 cells *via* interaction with LDLR.

The intracellular fate of EVs and their cargo internalized by LDLR-mediated endocytosis in PC-3 cells is closely related to their functionality. It could be postulated that EVs internalized by LDLR are transported to endosomes, late endosomes, and eventually lysosomes in which vesicular components could be degraded or recycled (47). We observed an accumulation of internalized EVs in perinuclear subcellular regions occupied in recipient PC-3 cells (Fig. 7*A*). Fluorescent imaging with ER or lysosome tracker staining revealed that intracellular EV signal largely overlapped with the lysosome and partially with ER (Fig. 7*D*). Given this localization, we reasoned that a subset of EVs transferred to the lysosome might have undergone proteolytic degradation suggested to have a trophic effect on recipient cancer cells (48). This may suggest that endocytic APOB-carrying EVs are rapidly degraded by recipient PC-3 cells, resulting in metabolic reprogramming to the Warburg phenotype.

## Discussion

We used an isogenic prostate cancer PC-3 cells and their acid-adapted PC-3AcT cells representing the Warburg phenotype with activated glycolytic activity by HK and PFKP activation but unchanged mitochondrial TCA cycle and OXPHOS (Fig. 1). In this study, we isolated EVs with a high confidence by combining SEC and ultracentrifugation. While PC-3AcT cells were hugely changed in their energy metabolism, the released concentration and size of EVs were not significantly affected (Fig. 1). However, quantitative proteomic analyses of these EVs showed significant regulation of proteins affected by metabolic changes of PC-3AcT cells according to lactic acidosis mimicking a low-oxygen tumor microenvironment. This changed landscape in PC-3AcT EVs included several novel observations, indicating a multifaceted functionality of EVs such as molecular regulation of EV biogenesis, their protein composition, uptake efficiency by recipient cells, and possible functional activity relating in metabolic reprogramming to Warburg phenotype.

At the cellular level, PC-3AcT cells showed the Warburg phenotype with differential expression of cellular metabolic enzymes including HK and PFKP with higher activity of HK to stimulate glycolysis, resulting in increased glucose consumption and ATP generation (Fig. 4). Transcriptomic analyses between PC-3 and PC-3AcT cells identified 962 regulated mRNA transcripts from a total of 13,683 protein-coding transcripts that are associated with cell adhesion and signal transduction (supplemental Fig. S1 and supplemental Table S1). Notably, about 6000 SNPs have been mutated from the parental PC-3 cells. Genomic instability is a key feature of prostate cancer, associated with tumor progression, resistance to cancer therapies, and metabolic reprogramming to the Warburg phenotype (49, 50, 51). Also, these accumulated genetic mutations regulate oncogenic signaling pathways and the activation of transcription factors such as HIF1A, conferring a survival advantage by facilitating rapid cell proliferation and adaptation to the hypoxic tumor microenvironment (49). Notably, these prostate cancer cells frequently exhibit the Warburg phenotype, characterized by elevated glycolysis and lactate production even in the presence of adequate oxygen. Although we were unable to address the genetic instability of acid-adapted PC-3AcT cells derived from parental PC-3 cells in this study, it would be necessary to investigate the involved molecular mechanisms in future study.

In the proteomes, these glycolytic enzymes were evidently observed in both EVs. PC-3 and PC-3AcT EVs are equipped with most of the glycolytic enzymes including GPI, ALDOA, TPI, GAPDH, PGK, PGAM, ENO1, PKM, and LDH (Fig. 3). This observation is consistent with previous EV proteomics studies that have confirmed the existence of glycolytic enzymes in EVs as annotated in the Vesiclepedia database (35) (supplemental Table S3). Metabolic enzymes in EVs and their functionalities have been proven in previous studies, reporting that these metabolic enzymes such as PKM2, ALDOA, and ALDH3A1 could be transferred to recipient cells by EVs to stimulate glycolytic metabolism of recipient cells (13, 14). Prostate cancer cells can also release PKM2-carrying EVs to educate the bone marrow environment into a metastatic niche by transferring PKM2 to bone marrow stromal cells with subsequent upregulation of CXCL12 cytokine (39). Another study has reported that hepatocarcinoma cells can actively release ENO1-carrying EVs targeting less-malignant hepatocarcinoma cells to promote a malignant transformation *via* increased expression of integrin α6β4 for metastasis (40). In this regard, vesicular glycolytic enzymes could be transferred by EVs into recipient cells involved in cancer progression.

Our quantitative proteomics showed similar levels of glycolytic enzymes in both EVs but more potent functionality in PC-3AcT EVs in the aspect of Warburg phenotype transition of recipient PC-3 cells. Reprogrammed PC-3 cells by PC-3AcT showed increased cellular proliferation and glycolytic energy metabolism as in their acid-adapted PC-3AcT cells. This metabolic reprogramming might be mediated by the overexpression of HK and PFKP with increased glucose consumption and ATP generation in reprogrammed cells educated by PC-3AcT EVs (Fig. 4). We postulate that this phenotypic transition of PC-3 cells might be closely related to their increased uptake of PC-3AcT EVs. When we treated PC-3 cells with fluorescent-labeled EVs, PC-3AcT EVs were significantly more taken up by PC-3 cells than PC-3 EVs. In this regard, our quantitative proteomics showed significantly increased expression of apolipoproteins such as APOB in PC-3AcT EVs. These apolipoproteins formed a physical interaction network with other proteins related to endocytosis and vesicle trafficking (Fig. 6). Importantly, APOB could be recognized by the LDLR of recipient cells and taken *via* receptor-mediated endocytosis. A well-known notion is that APOB is a representative lipoprotein in low-density lipoprotein. In the plasma, this is frequently identified in blood EVs in various studies with criticism as main contaminant from low-density lipoprotein during EV purification (52). However, some studies have identified apolipoproteins in EVs (45, 46). From our EV proteomics, APOB was identified by peptides of both translated and untranslated sequences in apolipoprotein B-48 (data not shown), indicating that apolipoprotein B-100 could be equipped in EVs.

While diverse roles of EV-mediated intercellular communication in the biology of tumor microenvironment have been widely investigated, the respective contribution of APOB-carrying EV for the Warburg phenotype is still poorly understood. Our observation of the upregulation of APOB in PC-3AcT EV raised a possibility that EV uptake through endocytosis was mediated by the LDLR of recipient cells. This uptake may play a role in the metabolic reprogramming of recipient prostate cancer cells. Whether the impact of EV engulfment entails a re-utilization of EV-associated molecules, as described earlier (42, 53), or other effects remain to be fully elucidated in the aspect of specific EV subtypes or their different subcellular destination (*i.e.*, endosome, ER. nucleus, or lysosome). Since PC-3 cells have some intracellular EV-related fluorescence mainly colocalized with the lysosome (Fig. 7), APOB-carrying PC-3AcT EVs might be trafficked to endosome and then preferentially to the lysosome later either intact or upon modification for metabolic reprogramming. Some taken EVs were also colocated in ER fluorescent regions by endosomal escape or re-emission with potential different biological activity. As mentioned earlier, our observations suggest that the functional endocytosis pathway plays a notable role in the capacity of acidic-adapted PC-3AcT cells to emit functional EVs to transit recipient cells to activate glycolysis. If so, modulating the uptake ability of cancer cells for EV utilization in tumor microenvironment may provide a way to develop innovative anticancer therapies.

It could be speculated that the elevated cellular proliferation for adaptation in an acidic microenvironment may require lots of molecular cargoes available through the uptake or reuptake of EVs (54), along with diverse functionality including cellular reprogramming. This is an interesting point in that the Warburg phenotype can increase tumor survival which is the major determinant in diverse tumor progression related to metastasis, angiogenesis, immunosuppression, and drug resistance in cancer pathogenesis (12). Especially, the Warburg phenotype is closely related to the resistance of cancer cells to chemotherapy associated with increased aerobic glycolysis in which upregulation of key enzymes of glycolysis, including HK, PKM, PDH, and LDH, contributes to tumorigenesis and chemoresistance (55). This metabolic transition of cancer cells provides an opportunity for drug resistance (56). Especially, HK, which is upregulated in reprogrammed PC-3 cells by PC-3AcT EVs, is the first rate-limiting regulator in the glycolytic pathway. Upregulation of HK is known to inhibit mitochondrial apoptosis by direct insertion of this enzyme into the mitochondrial outer membrane (57). Direct interaction of the voltage-dependent anion channel with mitochondrial-bound HK can inhibit cytochrome c release and subsequent cellular apoptosis (58). Apolipoprotein APOA1 and APOE-carrying EVs are also increased from nonsmall cell lung cancer cell A549 treated with cisplatin, a platinum-based alkylating agent that binds to DNA and inhibits replication of cancer cells (59). Although we did not address the aspect of metabolic reprogrammed cancer cells in drug resistance, it would be valuable to test the transfer of drug resistance property by EVs in metabolically reprogrammed cancer cells (9).

Our finding indicates that approximately 55% of PC-3AcT EVs were taken up by PC-3 cells for 7 h (supplemental Fig. S6). In our experimental condition, since 1 × 10^9^ of PC-3AcT EVs were treated to 100,000 of PC-3 cells, single PC-3 cell could take the about 10,000 of PC-3AcT EVs for 7 h. In our previous research (59), nano-flow cytometry analyses of single EV revealed that single A431-derived EV contains 254 molecules of CD63 and 513 molecules of tissue factor. If HK is present in approximately 100 molecules in an EV as speculation, a single PC-3 cell could take 1,000,000 of HK from 10,000 of PC-3AcT EVs. Madhukar *et al.* revealed that a single cancer cell contains from 100,000 to 1,000,000 molecules of HK (60). Although we do not know the recycling efficiency of EV proteins from lysosomal degradation to the cytosol, the HK molecules taken up by cells could potentially be functional if they traffic to the cytosol with relevant efficiency (61). Likely, the transfer and functionality of enzymes by EVs have been validated in the Cre-loxP method for studying EV transfer by Cre recombinase in the EVs (62).

Transcriptomics between PC-3 cells and PC-3AcT EV-treated PC-3 cells suggest that PC-3AcT EVs induced secondary changes in protein synthesis in recipient PC-3 cells. As evidenced by the upregulation of glycolysis-related mRNAs and the modulation of key metabolic pathways (Fig. 5), the proteins and RNA delivered by the PC-3AcT EVs likely initiate transcriptional and translational responses in the recipient PC-3 cells (63). This is consistent with previous findings by Al-Nedawi *et al.*, who demonstrated functional transfer of EGFR through glioma-derived EVs, and Valadi *et al.*, who showed the functional transfer of mRNA and miRNA by EVs (64). This suggests that EVs not only provide direct protein content but also influence the molecular machinery of the recipient cell, resulting in changes in gene expression and protein synthesis. However, the functional role of specific EV components, including the precise mechanisms through which these proteins such as functional enzyme, mRNA, miRNA, or other EV components are processed and utilized after endocytosis, remains unclear in this study. Our transcriptomic analyses of PC-3 cells treated by PC-3AcT EVs (Fig. 5) provide the clue that the metabolic reprogramming is not solely due to the direct incorporation of EV proteins into the cell. Instead, EVs act as carriers of signaling molecules, enzymes, and RNA that trigger downstream molecular changes in recipient cells. For example, the upregulation of glycolysis-related genes, such as SLC2A1 (GLUT1), in PC-3AcT EV-treated PC-3 cells suggests that EVs activate important metabolic pathways rather than simply providing additional proteins. These pathways likely result in significant cellular adaptations to the acidic tumor microenvironment and contribute to tumor progression through metabolic reprogramming.

Moreover, the efficiency of protein recycling and their subsequent functionality within the recipient cells, especially following their escaping from lysosomal degradation to the cytosol, requires further investigation. Additionally, our study mainly focuses on EVs derived from acid-adapted PC-3AcT cells, as the functionality of autologous PC-3 EVs was not effective at normal concentration during their culture. However, since EVs from different cellular status or origin may exhibit significant variations in cargo composition and functional properties. This highlights the need for further studies comparing EVs from different sources, including autologous and heterologous EVs, to better understand their diverse roles in metabolic reprogramming.

**Fig. 8:**
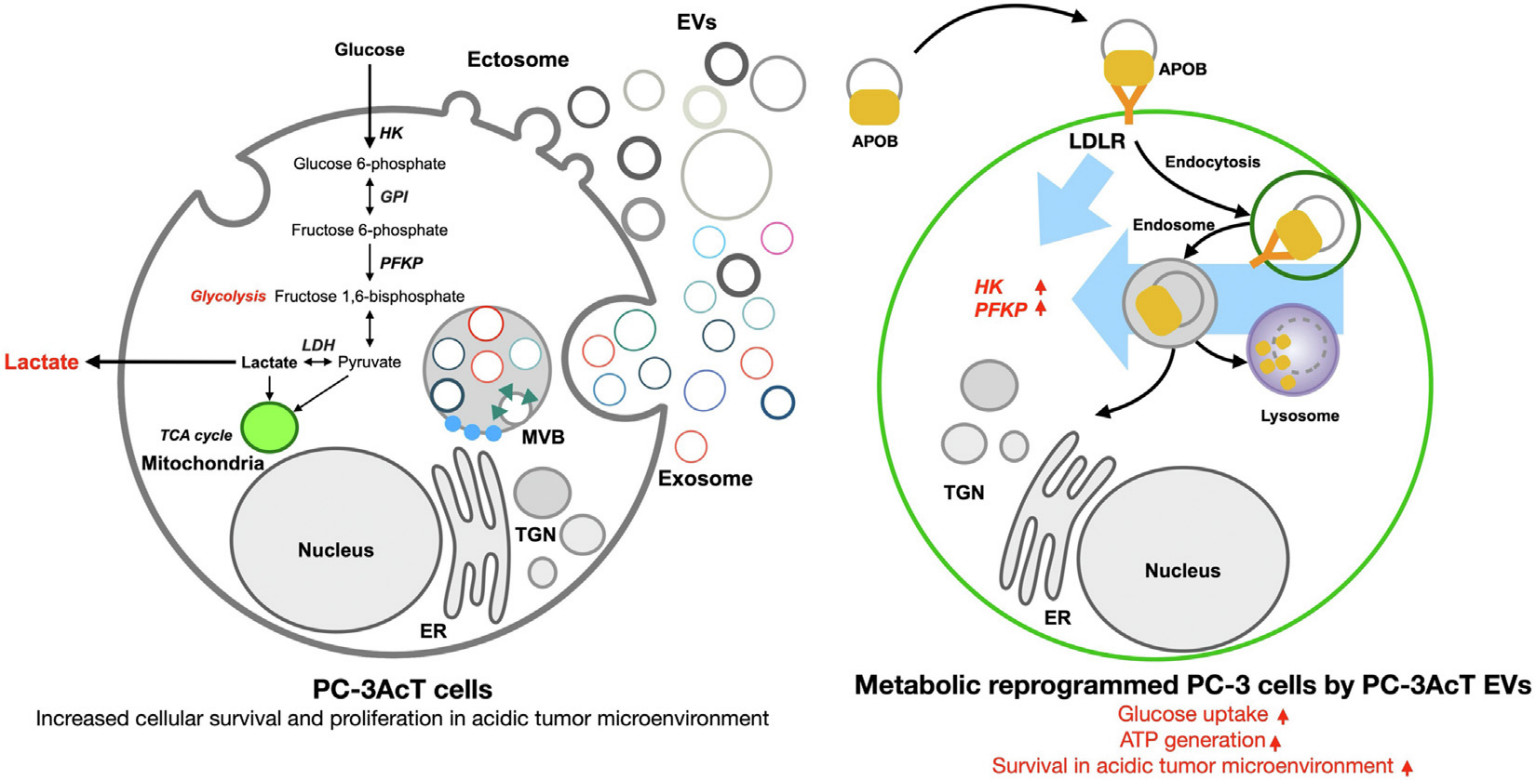
**A scheme for energy metabolic reprogramming of prostate cancer PC-3 cells mediated by PC-3AcT EVs.** PC-3AcT EVs could reprogram recipient PC-3 cells taken by vesicular APOB recognition by cellular LDLR, resulting in the activation of HK and PFKP to promote the activation of glycolysis favoring ATP generation caused by increased glucose consumption.

Overall, our results suggest that EV-mediated intercellular communication can regulate energy metabolic reprogramming in a low-oxygen tumor microenvironment. By applying quantitative proteomic analyses, we were able to show that regulating EV proteins affected by acidic stress is related to the functionality to transform recipient cells into Warburg phenotype *via* APOB-LDLR–mediated endocytic EV uptake. PC-3AcT EVs could reprogram recipient PC-3 cells by activating HK and PFKP to promote the activation of glycolysis favoring ATP generation caused by increased glucose consumption (Fig. 8). This energy metabolic reprogramming of PC-3 cells can result in increased survival and proliferation with levels similar to acid-sensitive PC-3AcT cells. In light of our observations, EV-mediated intercellular communication regarding energy metabolism in the context of extracellular acidosis is emerging as important knowledge to understand the tumor microenvironment as a mutual communication among heterogeneous cells. Our study first revealed that EVs derived from adapted prostate cancer cells in an acidic environment could transfer their energy metabolic property by EVs to recipient cells and that this acquired metabolic phenotypic transition favored cellular survival of recipient cells. Consequently, the acidic tumor microenvironment can impact the proteomic repertoire of EVs with significant implications for EV-mediated biological effects. Also, our findings highlight the importance of validating EV-mediated metabolic reprogramming *in vivo* within heterogeneous prostate cancer microenvironments, where intercellular communication through EVs plays a crucial role in tumor progression and therapy resistance. Understanding how EV-driven metabolic adaptations *in vivo* will be vital for developing targeted clinical strategies to disrupt these processes and improve treatment outcomes.

## Data Availability

All proteomic datasets including raw files and Scaffold file have been deposited to the ProteomeXchange Consortium *via* the PRIDE partner repository with the dataset identifier PXD054467. All transcriptomic datasets have been deposited to the NCBI Gene Expression Omnibus (GEO) database with accession number GSE283647.

## Supplemental data

This article contains supplemental data.

## Conflict of Interest

The authors declare no competing interests.
