# Supplementary material for: Metabolic Reprogramming Into a Glycolysis Phenotype Induced by Extracellular Vesicles Derived From Prostate Cancer Cells
Source: Mol Cell Proteomics. 2025 Mar 13;24(4):100944. doi: 10.1016/j.mcpro.2025.100944 (PMC12008616; doi:10.1016/j.mcpro.2025.100944)
Supplement: Supplementary Figures [file mmc2.pdf]

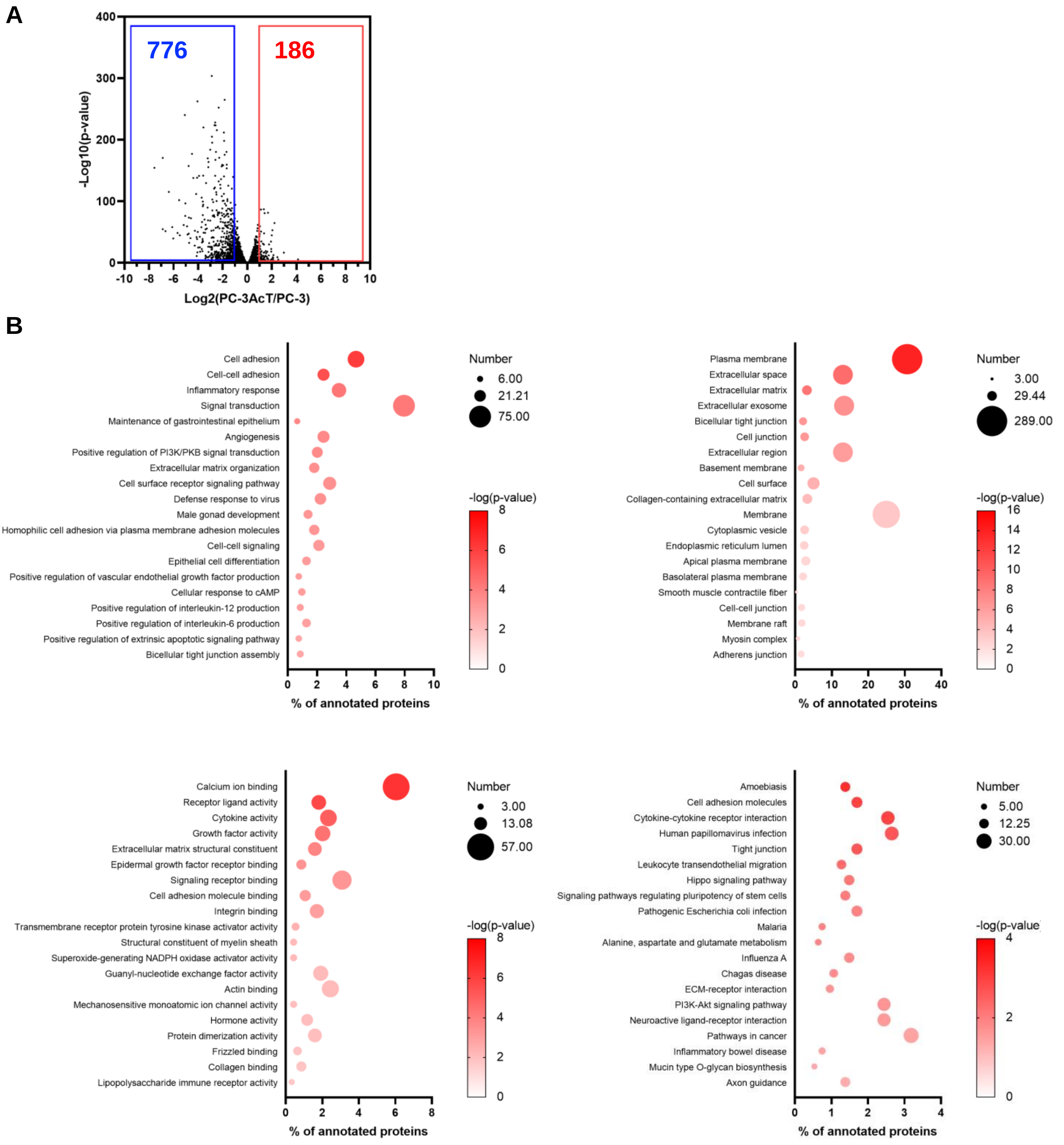

Fig. S1. Transcriptomics of PC-3 cells and PC-3AcT cells. (A) Transcripts of PC-3 and PC-3AcT cells were sequenced using NovaSeqX as described in the experimental procedures. Significantly altered mRNA were selected with a fold change greater than 2 and a *p*-value less than 0.05. In volcano plots, significantly downregulated mRNA were indicated with blue numbers, and upregulated mRNA with red numbers. (B) A total of 962 significantly regulated proteins were analyzed by Gene Ontology and KEGG pathway analysis using the DAVID bioinformatics database (davidbioinformatics.nih.gov/).

A

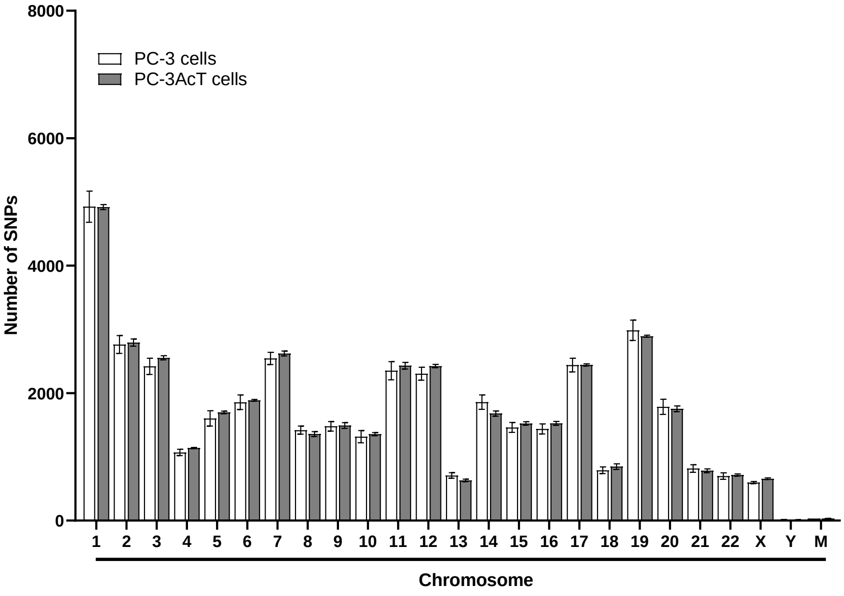

B

Chromosome	Number of different SNPs between PC-3 and PC-3AcT cells
1	637
2	337
3	318
4	138
5	191
6	234
7	333
8	202
9	186
10	158
11	350
12	309
13	210
14	335
15	217
16	235
17	328
18	122
19	548
20	231
21	133
22	121
X	100
Y	0
Mitochondrion	3

Fig. S2. Variant calling analysis for single nucleotide polymorphisms (SNPs) between PC-3 cells and PC-3AcT cells. (A) Histogram represents the number of SNPs in PC-3 and PC-3AcT cells in comparison to the reference sequence. (B) Table presents the number of additional mutations in PC-3AcT cells comparing with parental PC-3 cells. It is noteworthy that PC-3AcT cells harbor approximately 6,000 additional SNPs from parental PC-3 cells.

A

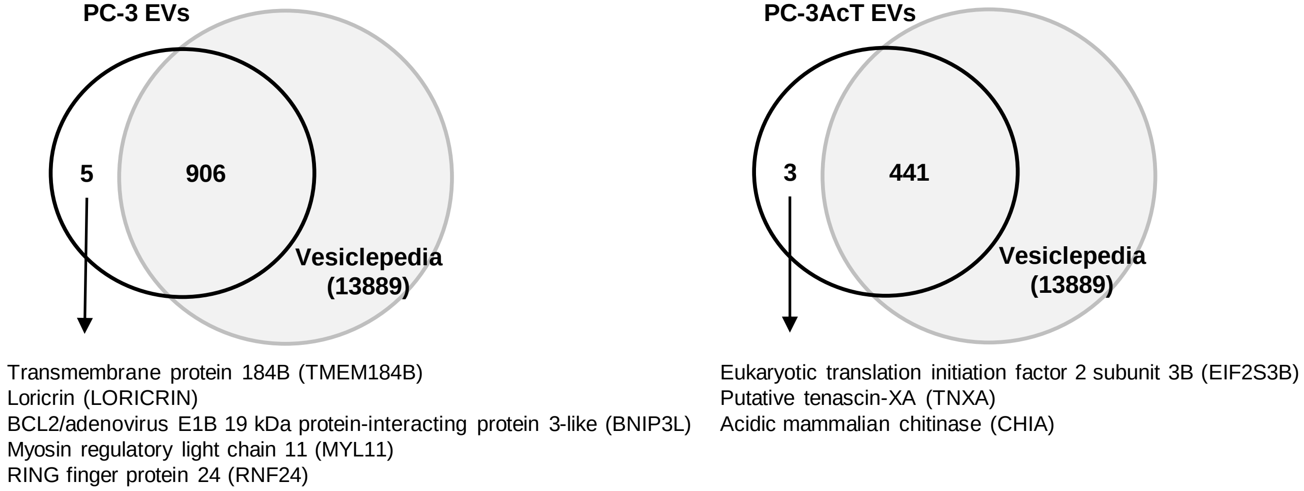

B

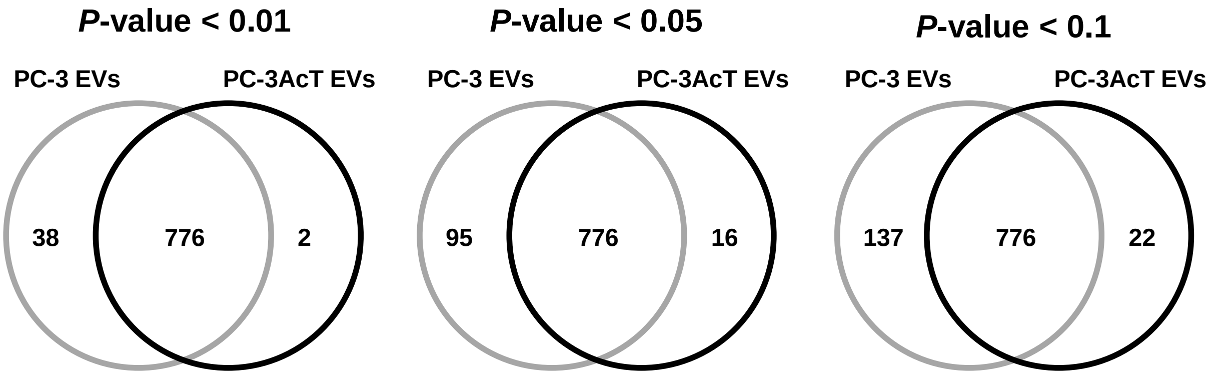

Fig. S3. Quantitative proteomics of PC-3 and PC-3AcT EVs. (A) Comparison of identified proteins in PC-3 EVs and PC-3AcT EVs with Vesiclepedia (www.microvesicles.org). Venn diagram indicated that the majority of both EV proteins were previously known EV proteins. (B) Ven diagram showed the differentially regulated proteins in PC-3 EVs and PC-3AcT EVs according to *p*-value.

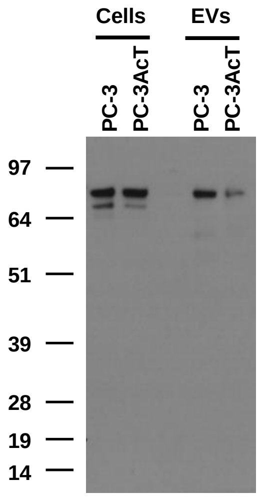

Fig. S4. Western blotting analysis of CTNNB1 from an independent biological replicate. The Western blotting confirms the differential expression of CTNNB1 as identified in proteomics. CTNNB1 was further validated in independently isolated EVs, as shown in Figure 2E.

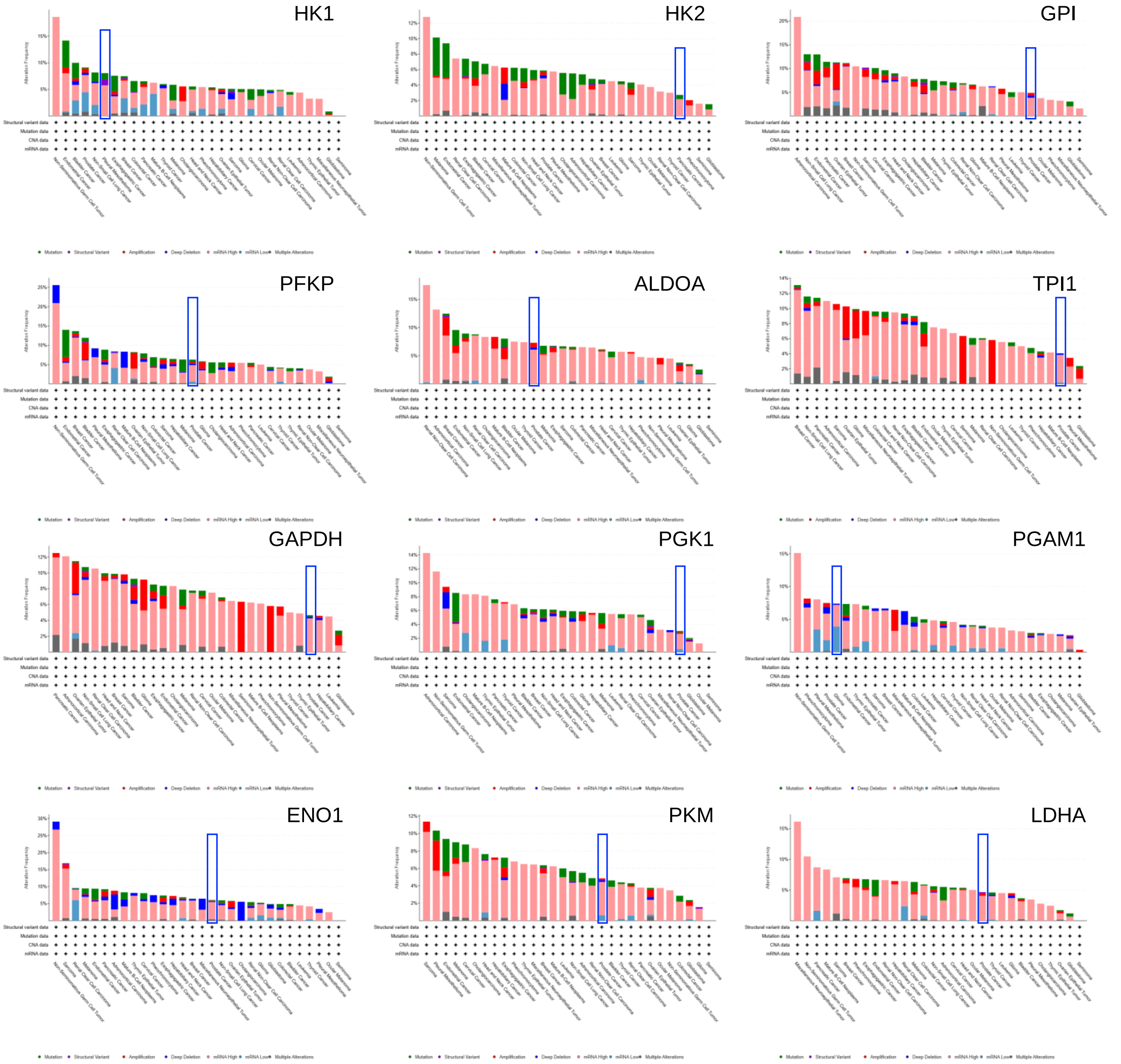

Fig. S5. Analysis of alterations frequency for glycolysis-related genes from TCGA PanCancer Atlas database. Glycolysis-related genes were analyzed in TCGA PanCancer Atlas database visualized using cBioPortal (cbioportal.org). The database comprising 10,967 samples from patients, providing the various cancer type information on structural variants data, mutation data, copy number variations and mRNA expression associated with glycolysis. Prostate caner category is indicated with blue box.

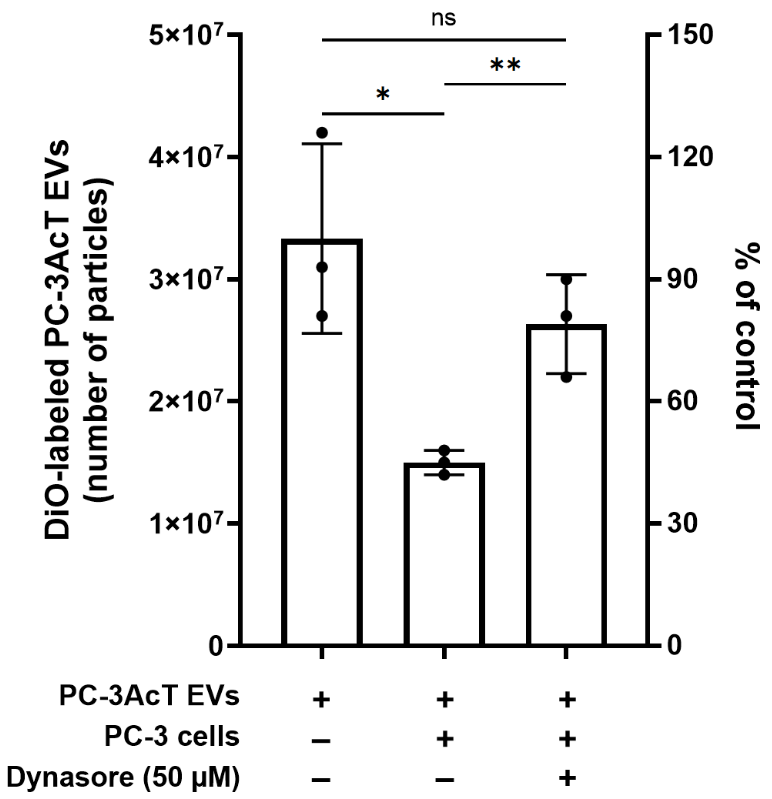

Fig. S6. Residual DiO-labeled PC-3AcT EVs of conditioned media in PC-3 cells with the presence of PC-3AcT EVs. PC-3 cells were seeded into 12-well plate at a density of 100,000 cells per mL of culture media and incubated for 24-h. Subsequently, a total of 1×10^9 PC-3AcT EVs were treated to PC-3 cells in 1 mL of culture media for 7-h. Approximately 8.41×10^7 DiO-labeled EVs were present in 1×10^9 PC-3AcT EVs. After 7-h of EV treatment, the conditioned media was collected and residual EVs were isolated by size exclusion chromatography, as described in the experimental procedures. Only DiO-labeled EVs were quantified using NTA in fluorescent mode. Comparing the input amount of DiO-labeled PC-3AcT EVs, approximately 45% of DiO-labeled EVs remained, suggesting that around 55% of EVs were taken or processed by PC-3 cells during the 7-h treatment period. Treatment of dynasore to PC-3 cells significantly inhibited the uptake of DiO-labeled PC-3AcT EVs, resulting in an increase in the residual DiO-labeled PC-3AcT EVs in conditioned medium.
